# A Multiscale Survival Process for Modeling Human Activity Patterns

**DOI:** 10.1371/journal.pone.0151473

**Published:** 2016-03-29

**Authors:** Tianyang Zhang, Peng Cui, Chaoming Song, Wenwu Zhu, Shiqiang Yang

**Affiliations:** 1 Department of Computer Science and Technology, Tsinghua University, Beijing, China; 2 Department of Physics, University of Miami, Miami, Florida, United States of America; Technical University of Denmark, DENMARK

## Abstract

Human activity plays a central role in understanding large-scale social dynamics. It is well documented that individual activity pattern follows bursty dynamics characterized by heavy-tailed interevent time distributions. Here we study a large-scale online chatting dataset consisting of 5,549,570 users, finding that individual activity pattern varies with timescales whereas existing models only approximate empirical observations within a limited timescale. We propose a novel approach that models the intensity rate of an individual triggering an activity. We demonstrate that the model precisely captures corresponding human dynamics across multiple timescales over five orders of magnitudes. Our model also allows extracting the population heterogeneity of activity patterns, characterized by a set of individual-specific ingredients. Integrating our approach with social interactions leads to a wide range of implications.

## Introduction

Human activity pattern is one of the central building blocks of modeling and understanding social dynamics such as information spreading [1–3], social-tie and group formations [4–7], social cooperations and competitions [8, 9]. While a wide range of social interaction models exist, they mostly assume that the communications among individuals are largely random, following a Poisson process. Yet, recent researches on human dynamics have demonstrated extensive evidence [10–12] that the interevent time (time between consecutive messages) and the response time (time between a message was received and the reply was sent) *τ* are heavy-tailed distributed, in contrast to prediction of the uncorrelated Poisson process where the interevent time distribution *P*(*τ*) follows an exponential form. This indicates that the vast majority of responses were sent within a very short time frame known as bursts. In some cases, however, the response stalls for a long time, as predicted by the long waiting times at the tail of the distribution. Specific examples range from communications [13, 14], entertainment [15, 16] and work patterns [17–19] to neural activities [20, 21], implying that there exists intrinsic complexity at each individual level even without involving social interactions. In other words, in social systems the “propagator” (interevent time distribution) *P*(*τ*) of a free “particle” (person) is fundamentally distinct from these in the physical science that are purely random (Guassian or exponential). In contrast, the intrinsic complexity of human behaviors such as long-memory effect are encoded and translated into the non-trivial form of *P*(*τ*), which significantly impacts on social dynamics at a macroscopic level. For instance, it has been subsequently shown [22] that the non-Poisson nature of the contact dynamics fundamentally alters spreading processes on networks, resulting in notably larger decay times than predicted by Poisson processes.

To capture underlying complexity in human activity patterns, various models have been developed during the past decade. Overall these models fall into two major approaches. The first approach mainly focuses on the microscopic foundation of human dynamics and tends to model how individuals make actions or responses. For instance, Barabási proposed a simple queuing model that allows to capture some essential ingredients of bursty dynamics [23–26]. These models mostly predict a power law *P*(*τ*) with universal exponent 1 and 1.5 for fixed and variable queues, respectively. The second approach, however, tends to model *P*(*τ*) directly without involving microscopic information at the individual level. Candidate models include Weibull distribution, log-normal distribution and Pareto distribution that follows power law for all *τ* greater than a threshold *x*_*m*_ [27–29]. These models have more practical flexibility compared to queuing models yet lack microscopic understandings of human dynamics.

**Fig 1:**
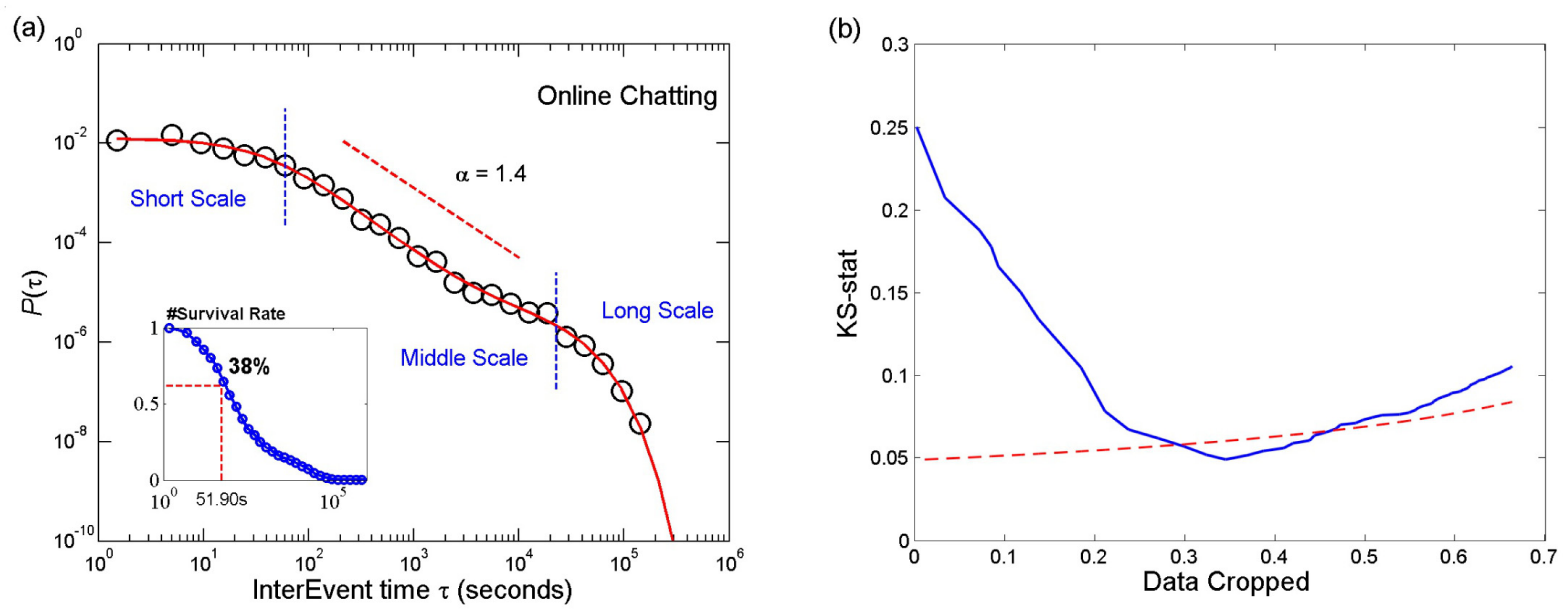
(a) The probability density function of the time interval between any user’s two consecutive messages in online chatting. *P*(*τ*) is well approximated by a power-law *τ*^−*α*^ with the exponent *α* = −1.4 for the intermediate time regime *τ* ∈ [10^2^, 10^4^] whereas for both shorter and longer time scales the power-law characteristic does not work. Left bottom inset: survival rate of the time interval. (b) When fitting with Pareto distribution, we discard the data with interevent time less than *x*_*m*_. We tune the parameter *x*_*m*_ and find the best fitting. It shows the relation between the fraction of cropped data less than *x*_*m*_ and the goodness of fit, which is measured by the KS statistic. The red line shows the threshold statistic to pass the KS test with the significance level of 5%, which gradually increases due to the decreased amount of data.

To demonstrate the challenges of modeling human activity pattern precisely, we plot *P*(*τ*) in Fig 1a for one online chatting user where *τ* is the interevent time of two consecutive messages (see Datasets section for details). The interevent time *τ* ranges over five orders of magnitudes and there is no simple distribution being able to approximate *P*(*τ*) across the entire time scale. For instance, a power law only captures an intermediate time regime *τ* ∈ (10^2^, 10^4^) approximately, and one has to crop data to perform power law fitting [30]. To quantify the effects of cropping, we fit *P*(*τ*) with the Pareto distribution for the same user which discards interevent time *τ* smaller than a parameter *x*_*m*_. Fig 1b shows the fraction of cropped data versus fitting goodness, showing that the power law fits well only after cropping 30% of the data. More severely, when apply the Pareto distribution to the population, less than 10% of the users pass statistical test even when croping 70% of the data. Fig 1 reveals the fact that there exist different time scales where individuals’ actions and responses have remarkably distinct patterns. This finding calls the needs for generic yet accurate models that enable to capture and quantify human dynamics across full-time scale. In this work, we report such a generic framework through a multiscale survival process.

## Materials and Methods

### Datasets

Our data-driven approach relies on accessibility of the following large-scale datasets of human activity pattern.

*Online chatting*: This data is collected from Tencent QQ, an MSN-style instant message platform in China, covering over 600 million users. We collect the log of users’ group chatting behavior that contains 50,000 online groups with 5,549,570 group members and the message log of each group during a 2 month period. The data records a unique user ID for every individual user and all timestamps when the user posts messages, which allows to construct each user’s activity pattern. All data is collected by Tencent QQ for academic research according to its terms and conditions. Furthermore, the data is fully anonymous and contains no identifiable information.

We also apply our method to the following two well-studied small-scale datasets for comparison and cross-validation:

*Letter correspondence*: The dataset is collected in the same way as in [24], and we adopt the similar analysis strategy. We collect 28511 letters of Einstein and 6944 letters of Freud, apart from the ones missing date or sender/receiver. We calculate the response time of the letters and analyze the distribution of it.

*Emails*: The dataset contains 3188 users and 129 135 records of sending and receiving Emails during a 3 month period in a university environment [10]. We analyze the distribution of time intervals between an individual sending two consecutive e-mails as in [12, 24].

### Model

The fact that the interevent time distribution *P*(*τ*) behaves differently at different time scales indicates that the intensity rate λ for an individual to make an action or response varies with the time *τ*. Therefore instead of modeling *P*(*τ*) directly, we are aiming to model the intensity function λ(*τ*) that encodes all essential microscopic details of the underlying stochastic process. Survival analysis [31–33] allows to connect λ to *P*(*τ*) through following rate equation

$$\begin{array}{c} \frac{\partial S(\tau |\theta )}{\partial \tau }=-\lambda \left(\tau |\theta \right)S\left(\tau |\theta \right), \end{array}$$ (1)

where the survival function *S*(*τ*|*θ*) is the probability of a waiting time longer than *τ*, and {*θ*} are individual specific parameters. Solving Eq (1) leads to

$$\begin{array}{c} S\left(\tau |\theta \right)=\exp\left(-\int_{0}^{\tau }\lambda \left(t|\theta \right)dt\right). \end{array}$$ (2)

Given the waiting time distribution *P*(*τ*|*θ*), the survival function *S*(*τ*|*θ*) simply corresponds to its complementary cumulative distribution

$$\begin{array}{c} S\left(\tau |\theta \right)=\int_{\tau }^{\infty }P\left(t|\theta \right)dt. \end{array}$$ (3)

Combining Eqs (2–3) allows to predict

$$\begin{array}{c} P\left(\tau |\theta \right)=-\frac{\partial S(\tau |\theta )}{\partial \tau }=\lambda \left(\tau |\theta \right)\exp\left(-\int_{0}^{\tau }\lambda \left(x|\theta \right)dx\right). \end{array}$$ (4)

Here the *P*(*τ*|*θ*) is a generic form of several simple distributions commonly used to model human dynamics. A time-independent intensity rate λ corresponds to a homogeneous Poisson process, where Eq 4 simply recovers the well-known exponential waiting time distribution. If λ varies with time *τ*, we are able to achieve various *P*(*τ*) forms. For instance, λ(*τ*) = *γ*/*τ* leads to a power law waiting time distribution *P*(*τ*)∼*τ*^−(1+*γ*)^ whereas λ(*τ*) = *γ*/*τ*^*α*^ with an exponent *α* < 1 recovers the Weibull distribution. Nevertheless, as we discussed above, these simple forms capture only limited temporal regimes of the empirically observed *P*(*τ*).

To incorporate different activity patterns across multiple time scales, we propose the following generic intensity function

$$\begin{array}{c} \lambda \left(\tau |\theta \right)=\frac{\lambda_{0}}{{\left(\tau /t_{0}\right)}^{\alpha }+1}+\lambda_{\infty }, \end{array}$$ (5)

where *θ* = {λ_0_, *t*_0_, *α*, λ_∞_} captures the following different aspects in human activity patterns:

**λ**_**0**_ determines the activity rate in a small time scale. If *τ* ≪ *t*_0_, (*τ*/*t*_0_)^*α*^ ≈ 0 and λ_0_ ≫ λ_∞_, we find λ(*τ*) ≈ λ_0_. The larger λ_0_ is, the higher the probability that the user makes a quick response will be.

***t*** **_0_** determines the critical time scale where a highly heterogeneous activity pattern starts to emerge. This phenomenon is well-known as the burstiness of human dynamics [34, 35]

**α** > 0 controls the degree of the heterogeneity of burst regime.The larger *α* value leads to more heterogeneous activities raised from underlying human dynamics.

**λ**_**∞**_ determines the activity rate in a large time scale, e.g. lim_*τ* → ∞_ λ(*τ*) = λ_∞_. λ_∞_ ≪ λ_0_ so that λ_∞_ has little influence until *τ* is big enough. The exponential tail is greatly influenced by λ_∞_ and it also plays a leading role in the average time interval of human activities.

To learn our modelling parameters, we start from a set of empirical records of an individual’s interevent times *T* = {*τ*_1_, *τ*_2_, …, *τ*_*n*_}, and calculate the following likelihood function

$$\begin{array}{c} L\left(\theta \right)=\prod_{i=1}^{n}P\left(\tau_{i}|\theta \right)=\prod_{i=1}^{n}S\left(\tau_{i}|\theta \right)\lambda \left(\tau_{i}|\theta \right). \end{array}$$ (6)

The corresponding log-likelihood function reads

$$\begin{array}{c} \ln L\left(\theta \right)=\sum_{i=1}^{n}\left\{\ln\lambda \left(\tau_{i}|\theta \right)-\int_{0}^{\tau_{i}}\lambda \left(t|\theta \right)dt\right\}. \end{array}$$ (7)

Maximizing Eq (7) regarding {λ_0_, *t*_0_, λ_∞_, *α*} leads to estimated modeling parameters of the interevent times *T*.

## Results

**Fig 2:**
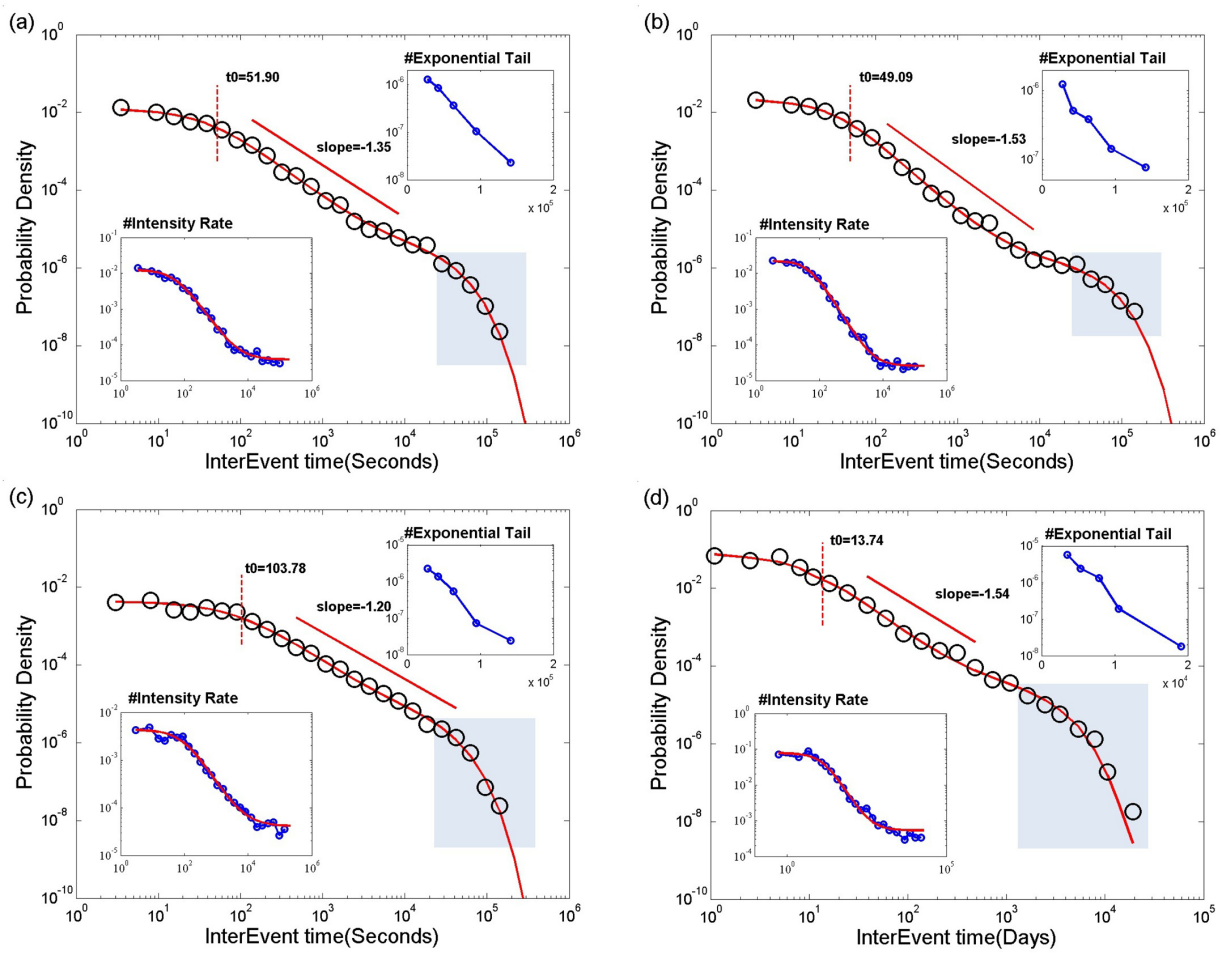
The interevent time distribution *P*(*τ*) for (a-b) two users from online chatting dataset, (c)one user for Email reply and (d) one individual for letter correspondence, respectively. The black circle represents the real data and the red curve is the model’s prediction. Right top inset: *P*(*τ*) in a semi-log plot. Left bottom inset: intensity rate λ versus waiting time *τ*.

Fig 2 demonstrates *P*(*τ*) for four randomly selected individuals across our datasets. Despite notable diversity over different individuals and datasets, our model excellently agrees with the empirical data across a full-time regime over five orders of magnitudes. In contrast, a power law fitting is often limited within an intermediate time-scale over 1–2 orders of magnitudes. The sharp cutoff at large time scale reveals a clear exponential tail in a semi-log plot in line with the prediction of our model (Fig 2 Inset). Note that a typical timescale of such cutoff (e.g. start around 6 hours for online chatting dataset) is much shorter than the duration of data records(two months in our case), implying that it is rooted in the intrinsic ingredients of human activity instead of a finite-size effect. We also find that the time-dependent intensity rates λ(*τ*) monotonically decease with *τ* that are very well captured by our model Eq (5).

To evaluate the performance of our model, we apply our methodology to 26,648 active users(with more than 100 records during two month periods) from the online chatting dataset and apply several standard statistical tests including Kolmogorov-Smirnov test (KS test), chi-square test and Cramér-von Mises test. We set the significance level to 5% to judge whether the fitting is good enough or not. In addition, we use the average statistic as metric, which represents the magnitude of error between real data and the fitting result of the interevent time distribution.

Table 1 shows the average statistic of KS-test, chi-square test and Cramér test and the pass rate with the significance level of 5% in these three statistical tests on online chatting dataset.

**Table 1:** Goodness of fit for the online chatting dataset, measured by the average statistic and pass rate with the significance level of 5% of three statistical testing method. Our model performs best in all metrics.

	KS test		chi-square		Cramer	
	stat	rate	stat	rate	stat	rate
Poisson	0.713	0.0%	8445.5	0.0%	79.43	0.0%
Pareto	0.281	0.5%	612.91	0.0%	19.91	0.5%
Weibull	0.202	1.7%	402.89	1.9%	5.46	2.0%
Log-normal	0.145	8.1%	216.24	3.7%	2.16	8.0%
Model	**0.070**	**70.6%**	**67.99**	**51.3%**	**0.66**	**76.54%**

As the table shows, our model beats all the baselines significantly in all metrics. 70.6% of users pass the KS-test with the significance level of 5%. If we lower the significance level to 1%, the pass rate will increase to 81.8% and it is quite an inspiring result. As for the bad case, we found that data sparsity is a main reason and observing more data will make the model more precise.

The improvement compared to baselines is mainly attributed to the truth that our model captures multi-time-scale characteristics of human dynamics in a more detailed and comprehensive way. The Poisson process performs the worst among all models since it cannot capture the heavy-tail feature of human dynamics. While Pareto distribution, Weibull distribution and log-normal distribution can capture heavy-tail feature, it only works for the middle time scale. As a result, if we try to fit the whole time scales of human dynamics, such models embody obvious limitation and are significantly exceeded by our model in all metrics. The high pass rate of our model also indicates that it captures multiple time scale human behaviour for real application.

**Fig 3:**
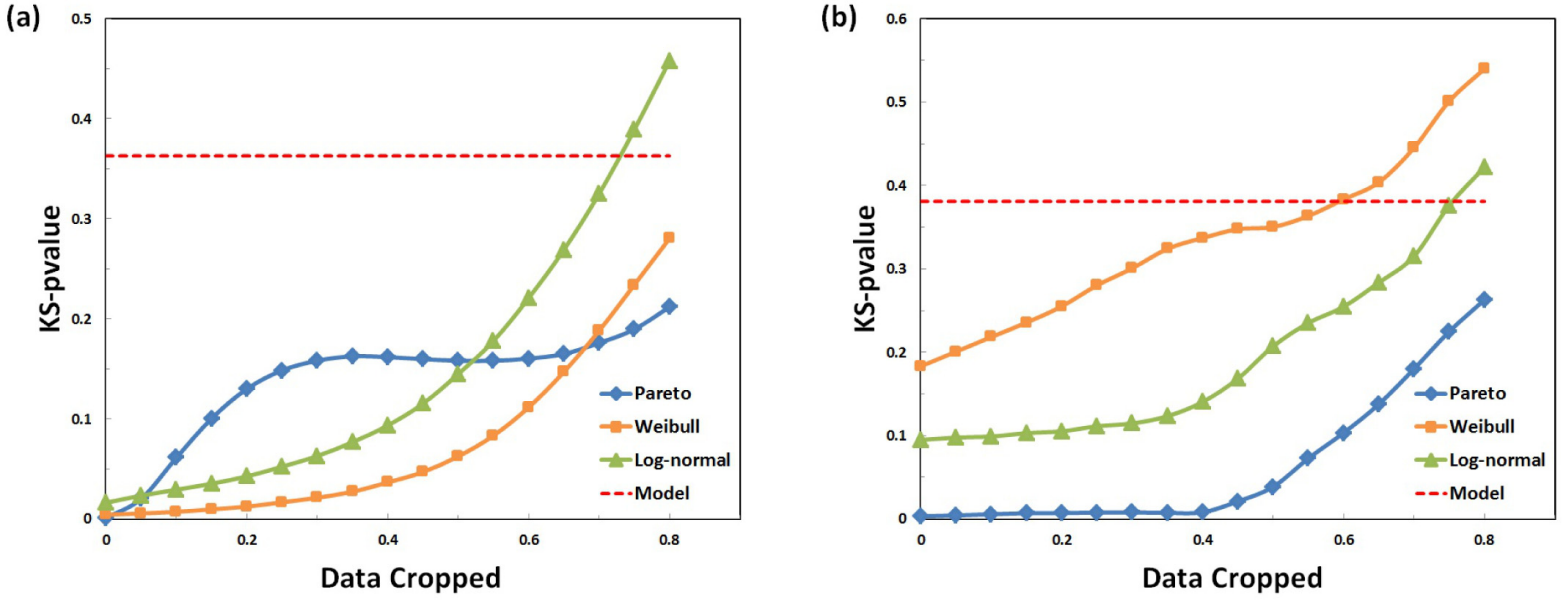
Average KS p-value after cropping, (a) online chatting dataset and (b) email activity dataset, respectively. The horizontal axis shows the fraction of data cropping. The vertical axis shows the average p-value in KS test, where higher value represents a better fitting. The blue, orange and green curves show the goodness of fit of Pareto distribution, Weibull distribution and log-normal distribution respectively. The red dotted line shows the p-value of our model without cropping data.

We also compare the goodness of fit under different time scales after cropping data. Fig 3 shows the result of average KS p-value after cropping the short interevent time data on online chatting dataset and email dataset. We can see that the goodness of fit (measured by average p-value of KS test) all increases when gradually cropping data and stays relatively steady after discarding about 30%—40% of the data. The Pareto distribution, Weibull distribution and log-normal distribution show different patterns, reflecting partial information of human dynamics. Pareto distribution performs worst for the full data yet improves significantly when cropping 30% of the data in short-time-scale. Log-normal distribution performs relatively stable and has advantage over Pareto distribution in shorter and longer time scales, implying that the early period and later period of human dynamics are non-power-law. The performance of our model (without cropping data) is represented by red dotted lines, showing remarkable improvements over all baselines.

**Fig 4:**
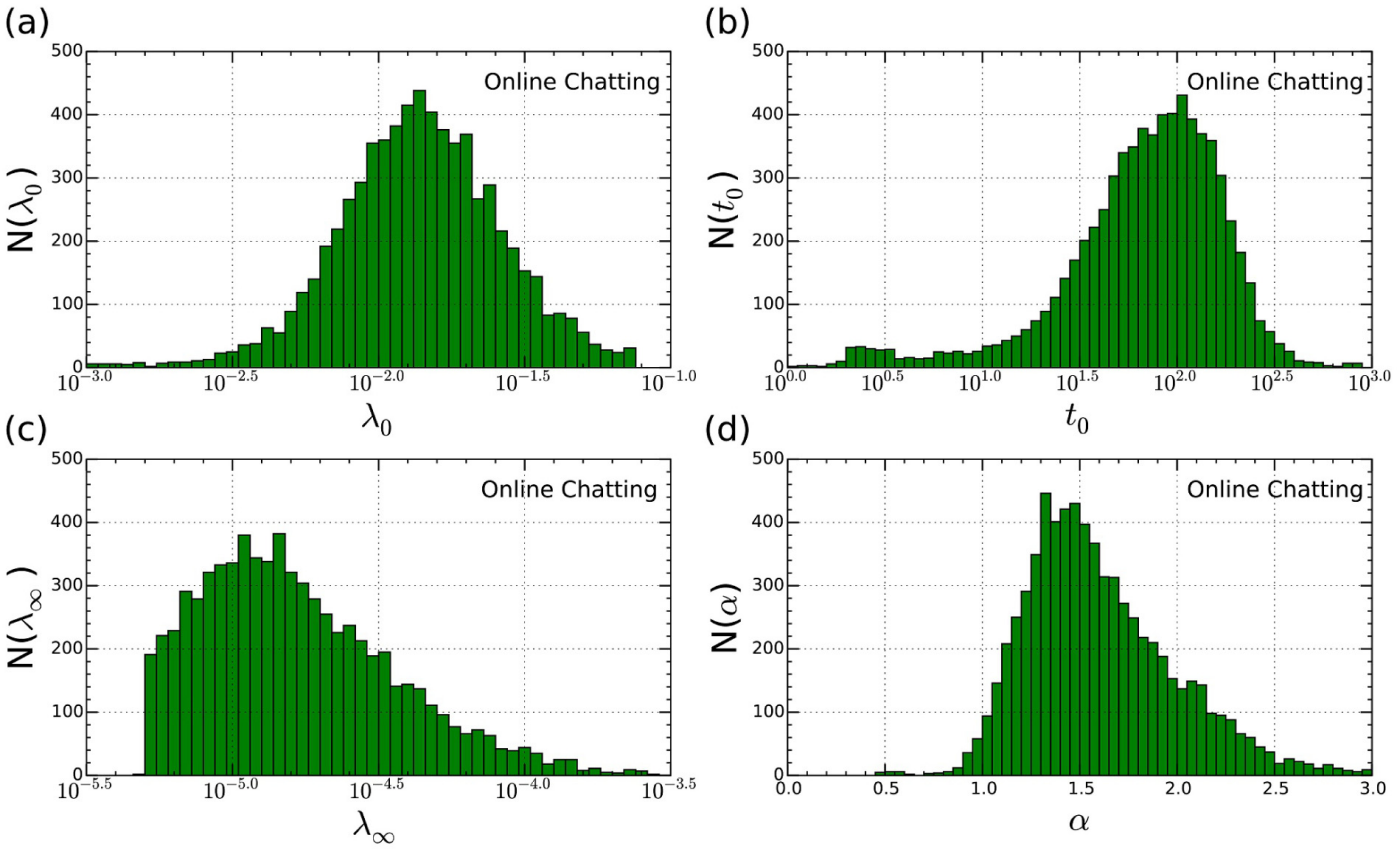
Distribution of parameter (a) λ_0_, (b) *t*_0_, (c) λ_∞_ and (d) *α* for the online chatting dataset, respectively.

Fig 4 plots the distributions of our modeling parameters across the online chatting dataset, finding that

**λ**_**0**_ follows a log-normal distribution quite well. Since it mainly influences the percentage of short interevent intervals, we may infer that people’s short time response patterns is stable around different users and the percentage of quick responses is also near Gaussian distribution;

***t***_**0**_ follows a skewed log-normal distribution with 100 seconds as the modal number. As *t*_0_ is the typical time scale of human activity between the quick response and bursty patterns, the plot indicates that for most people, the power-law distributed activity dynamics dominates after a relatively long period. We hypothesise that *t*_0_ corresponds a time scale that an individual sticks to a certain topic whereas for the *τ* > *t*_0_, the user starts to lose his/her interests and switch to other topics. Further studies need to be performed to test this hypothesis.

**λ**_**∞**_ has a strong correlation with the average time interval in human dynamics. It can be approximated by a log-normal distribution with a cutoff at small value, a fact due to the sampling bias of neglecting inactivity users.

***α*** follows a skewed normal distribution with an average value close to 1.45, in line with the prediction of queue models with various queue size [24].

## Discussion

To conclude, previous studies have shown that human dynamics is characterized by bursts of events and long periods of inactivity. Nevertheless, the nature of burst dynamics remains elusive. Our study of high resolution records of human interactive behavior provides an in-depth analysis of human dynamics, revealing non-Poisson temporal patterns that suggests a rethinking of mechanisms governing the human dynamics. Rather than focusing on limited time regimes, we find different patterns over multiple time-scales. We propose a generic model which not only captures the microscopic dynamics comprehensively but also predicts the interevent time distribution of each individual accurately. In this way, our model offers a generic modeling framework of the dynamics of human activities, potentially impacting a wide range of applications from marketing to education and politics.

## Supporting Information

S1 File A Multiscale Survival Process for Modeling Human Activity Patterns SI. (PDF)
