# Supplementary material for: A Multiscale Survival Process for Modeling Human Activity Patterns
Source: PLoS One. 2016 Mar 29;11(3):e0151473. doi: 10.1371/journal.pone.0151473 (PMC4811539; doi:10.1371/journal.pone.0151473)
Supplement: S1 File — (PDF) [file pone.0151473.s001.pdf]

A Multiscale Survival Process for Modeling Human Activity Patterns
Supplementary Information

Tianyang Zhang[1], Peng Cui[1,*], Chaoming Song[2,+], Wenwu Zhu[1], and Shiqiang Yang[1]

[1]Department of Computer Science and Technology, Tsinghua University
[2]Department of Physics, University of Miami
[*]corresponding.cuip@tsinghua.edu.cn
[+]corresponding.csong@physics.miami.edu

S1. event time

In real dataset such as E-mail and online group chatting, the distribution of real interevent time often appears a peak ranging from $\Delta t = 16$ hours to $\Delta t = 24$ hours. This phenomenon can be explained by sociological behavior involving the time (usually 16 hours) between when people leave work and when they return to their offices. In such situation, we often use event time instead of physical time to avoid the influence. Assume that we have all the records of users' behavior form time 0 to T , the time point of the behaviors of a certain user is denoted as $t_1, t_2, \ldots$. Define a function $N(t)$ that equals the total number of events of all the users(i.e., an message sent) before time t.

If the physical time is t_i, we define the corresponding event time t_i' as follows,

$$t_i' = \frac{N(t_i)}{N(T)} \cdot T \tag{1}$$

The time interval between t_i and t_j in event time is denote as follows,

$$\tau = t_j' - t_i' = \frac{N(t_j) - N(t_i)}{N(T)} \cdot T \tag{2}$$

Event time uses a "tick" of system (i.e., an message sent) as the basic time unit to uniform the time period.

S2. baselines

In the experiment, we compare our model with several baselines, they are defined as follows:

Poisson Process: The waiting time distribution of Poisson process is actually Exponential Distribution. The probability density function(*p.d.f*) is

$$P(\tau) = \lambda_0 \exp\left(-\lambda_0 \tau\right), \tag{3}$$

intensity function is

$$\lambda(\tau) = \lambda_0, \tag{4}$$

in which λ_0 is a parameter. We can estimate this parameter by maximum likelihood estimator (MLE) and the result is

$$\lambda_0 = n / \sum_{i=1}^{n} \tau_i. \tag{5}$$

Pareto Distribution: Pareto Distribution represents the power law feature. The probability density function(*p.d.f*) is

$$P(\tau) = \alpha \tau_m^\alpha / \tau^{\alpha+1}, \tag{6}$$

intensity function is

$$\lambda(\tau) = \alpha / \tau, \quad \tau > \tau_m, \tag{7}$$

in which τ_m and α are two parameters. Denote that Pareto Distribution requires that all interevent time $\tau > \tau_m$, so when used in real data, we often crop data and discard the data less than τ_m. The logarithmic likelihood function is

$$\ell(\alpha, \tau_m) = n \ln \alpha + n\alpha \ln \tau_m - (\alpha + 1) \sum_{i=1}^{n} \ln \tau_i, \tag{8}$$

so the parameter can be learned use maximum likelihood estimator (MLE).

Weibull Distribution: Weibull Distribution is a widely used and studied distribution, is has lots of modification form , here we use the basic model with two parameters. The probability density function(*p.d.f*) is

$$P(\tau) = \frac{\alpha \tau^{\alpha-1}}{\lambda_0^\alpha} \exp\left(-(\tau/\lambda_0)^\alpha\right), \tag{9}$$

intensity function is

$$\lambda(\tau) = \frac{\alpha \tau^{\alpha-1}}{\lambda_0^\alpha} \tag{10}$$

Log-normal Distribution: Log normal distribution is similar to normal distribution, the probability density function(*p.d.f*) is

$$P(\tau) = \frac{1}{\tau \sigma \sqrt{2\pi}} e^{-\frac{(\ln \tau - \mu)^2}{2\sigma^2}}, \tag{11}$$

in which μ and σ are two parameters. The estimation of parameter is similar to normal distribution, the result is as follows,

$$\hat{\mu} = \frac{\sum_i \tau_i}{n}, \tag{12}$$

$$\hat{\sigma}^2 = \frac{\sum_i (\ln \tau_i - \hat{\mu})^2}{n}. \tag{13}$$

S3. statistical tests

In experiment, we use three statistical tests as metrics of goodness of fit. We will give a brief introduction of each statistical test in the following:

Kolmogorov-Smirnov test: Kolmogorov-Smirnov test (KS test) is a nonparametric test of the equality of continuous, one-dimensional probability distributions that can be used to compare a sample with a reference probability distribution. The statistic is,

$$D_n = \sup_x |F_n(x) - F(x)| \tag{14}$$

where $\sup_x$ is the supremum of the set of distances. F_n is the empirical distribution function for n iid observations X_i, defined as,

$$F_n(x) = \frac{1}{n}\sum_{i=1}^{n} I_{[-\infty,x]}(X_i) \tag{15}$$

where $I_{[-\infty,x]}(X_i)$ is the indicator function, equal to 1 if $X_i \le x$ and equal to 0 otherwise.

Chi-squared test: Chi-squared test, also referred to as χ^2 test, is a statistical hypothesis test in which the sampling distribution of the test statistic is a chi-square distribution when the null hypothesis is true. The statistic is,

$$\chi^2 = \sum_{i=1}^{n} \frac{(O_i - E_i)^2}{E_i} \tag{16}$$

where O_i is the number of observations of type i, E_i is the expected (theoretical) number of type i.

Cramér–von Mises test: Cramér–von Mises test (Cramér test) is a criterion used for judging the goodness of fit of a cumulative distribution function F^* compared to a given empirical distribution function F_n. The statistic is,

$$T = n\omega^2 = \frac{1}{12n} + \sum_{i=1}^{n}\left[\frac{2i-1}{2n} - F(x_i)\right]^2 \tag{17}$$

where $x_1, x_2, \cdots, x_n$ are the observed values in increasing order.
